# Real-World Patient Experience With PrabotulinumtoxinA in the United Kingdom: A Single-Center Survey and Analysis of 254 Patients

**DOI:** 10.1093/asjof/ojae013

**Published:** 2024-02-27

**Authors:** Kathryn Taylor-Barnes

## Abstract

**Background:**

Botulinum toxin Type A (BoNT-A) injection is the most widely performed nonsurgical cosmetic procedure in the United Kingdom. PrabotulinumtoxinA is a new BoNT-A, recently licensed in the United Kingdom, for treating moderate-to-severe glabellar lines in adults under 65. Although clinical trials have established safety and efficacy, real-world data can help clinicians translate study findings into practice and support prabotulinumtoxinA use in a more diverse patient population.

**Objectives:**

To understand the real-world patient experience and patient perceptions of prabotulinumtoxinA performance for treatment of the glabellar region.

**Methods:**

In this single-center survey study, a single injector administered prabotulinumtoxinA for the treatment of glabellar lines to real-world patients presenting for BoNT-A treatment. Two weeks later, patients received surveys asking about their experience through email. There were no incentives for participation. Responses returned within 5 weeks of treatment were included.

**Results:**

From February to June 2023, 457 patients received prabotulinumtoxinA injections for glabellar line treatment. Survey response rate was 56% (254/457 patients). For most patients, treatment onset was 2 to 3 days following injection and peak response occurred after 7 to 10 days. Adverse effects were minimal, with 67% of patients experiencing none. Among survey respondents, 83% rated their treatment positively (5-point satisfaction scale), and 72% would choose prabotulinumtoxinA again.

**Conclusions:**

These data support safety and effectiveness of prabotulinumtoxinA in a diverse, real-world population, and confirm patient satisfaction among experienced BoNT-A patients, as well as suggest a rapid time to onset and peak effect.

**Level of Evidence: 4:**

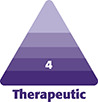

Treatment with botulinum toxin Type A (BoNT-A) is the leading nonsurgical cosmetic procedure performed in the United Kingdom and worldwide, accounting for nearly half of the nonsurgical procedures performed in 2022.^1^ Over the past 2 decades, various BoNT-A formulations, including onabotulinumA, incobotulinumA, and abobotulinumA, have been licensed for cosmetic applications in the United Kingdom.^2^ In 2023, a fourth BoNT-A toxin, prabotulinumtoxinA (Nuceiva [marketed as Jeuveau in the United States]; Evolus, Newport Beach, CA), was approved in the United Kingdom for the temporary improvement in the appearance of moderate-to-severe glabellar lines in adult patients <65 years of age.^3^

Importantly, the clinical trials, which led to the clearance of these BoNT-A products, were conducted in a highly controlled fashion: BoNT-A injection patterns, product dosage, and concentrations were standardized, and patients met strict inclusion and exclusion criteria.^4^ Although these features are important elements of controlled testing, in real-world practice, a much wider range of patients are treated (eg, patients over 65 years of age, those who have been treated with BoNT-A before, injection patterns are tailored to individual patients). Furthermore, in the case of BoNT-A toxin, clinical trials generally restrict product use to a single area, whereas real-world treatment often includes multiple areas, including off-label treatment areas.

Thus, translating clinical study findings into everyday clinical practice represents a persistent challenge.^4^ In contrast, real-world studies are intended to measure product efficacy and safety in all patients who would receive treatment as a part of their clinical care. These studies lack the tight control over experimental variables: there can be variations in treatment, there may be different endpoints, and real-world studies can also have challenges with data completeness; however, these studies serve an important function by confirming treatment performance in a wider population, in those patients actually receiving the treatment in everyday clinical care.^4-6^ In Supplemental Figure 1, the wide range of patient ages, dynamic line patterns, and line severity encountered in real-world clinical care are apparent. For BoNT-A, gathering real-world data can be challenging, because there are no formal registries to monitor real-world performance, nor are there independently funded real-world studies.

Although prabotulinumtoxinA has been evaluated in several Phase III, placebo-controlled clinical trials^7-11^ and post hoc analyses have demonstrated its efficacy and safety in various patient cohorts, including males,^12^ millennials,^9^ those over the age of 65,^11^ and those with skin color,^10^ real-world data are lacking because of the recency of approval. The objective of the present study was to understand the real-world patient experience and perceived performance of prabotulinumtoxinA for treatment of the glabellar region through a nonincentivized patient survey following injection.

## METHODS

This single-center injector, survey-based study evaluated product performance and patient experience with prabotulinumtoxinA for treatment of glabellar lines in the first 457 consecutive patients treated with prabotulinumtoxinA from February to June 2023. This study was not IRB approved, because it was originally designed as a patient experience survey. All patient care and interactions were carried out, according to the principles outlined in the Declaration of Helsinki.

### Treatment

Patients included were treated by a single injector within a single practice from February to June 2023. Injections were carried out using prabotulinumtoxinA. In all cases, a 50 U vial was reconstituted with 0.5 cc normal saline. All patients included in the analysis were treated in the glabellar area; however, patients were permitted to receive treatment in other areas of the face as well. Injection patterns and BoNT-A doses were customized to individual patient needs based on their anatomy and treatment areas included: there were no standardized injection patterns used. A video showing an example of injection technique is presented in the video, above.

### Data Collection

At the time of treatment, patients were informed that they would receive an email with a link to provide treatment and product feedback. Surveys were sent out to all patients 2 weeks after receiving treatment with prabotulinumtoxinA through email with a link to a Google Analytics form. Patients received no incentive for completing the survey. Patients were asked 12 questions regarding their clinical history, experience, effects with the treatment, satisfaction, and feedback (Supplemental Table 1). Patients could select predesignated answers or enter a customized response by selecting “other.” The included responses were returned within 5 weeks of treatment.

### Data Analysis

Survey responses to each question were analyzed. Any “other” responses that did not answer the question or were unclear were omitted from the analysis of that question, leading to slightly different response numbers for each question. More than 1 response for a single respondent was included, if appropriate (eg, adverse effects).

## RESULTS

### Demographics

A total of 457 patients were injected with prabotulinumtoxinA for treatment of glabellar lines from February 2023 to June 2023. The survey response rate was 56%, with 254 patients completing the survey. Twenty-four percent of those completing the questionnaire (61/254) were millennials (born 1981-1996), which was similar to the percentage of millennials treated overall (28%, 127/457). Survey respondents represented a wide range of ages (Supplemental Figure 1). Among those reporting their age (*n* = 212), the mean age was 49 (range, 21-77 years). The mean age for treated individuals was 48 years. All survey responses were returned between 2 and 5 weeks following injection.

### Clinical History

Most survey respondents (70%) had received an antiwrinkle injection treatment within 3 to 6 months before the evaluated prabotulinumtoxinA injection (Supplemental Figure 2). A total of 16% of patients received an antiwrinkle injection greater than 6 months prior, whereas 8% had been injected within 3 months. The evaluated prabotulinumtoxinA injection was the first antiwrinkle injection for 7% of the patients.

Seventy-six percent (194/254) of respondents were not taking other medications at the time of injection. Over a third of patients receiving medication at the time of injection were taking hormone replacement therapy (HRT; 22/60). Among the other commonly listed medications were statins, thyroxine, and HRT. Among respondents with a definitive date of receiving the COVID vaccine (165/254), 36% received it within 6 months of the prabotulinumtoxinA injection.

### Effect of Treatment

**Figure 1.**
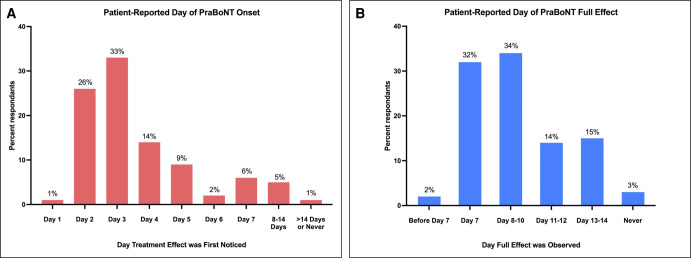
Patient-reported day of prabotulinumtoxinA treatment onset and full effect. (A) Plotted survey responses to “On what day after treatment did you first notice an effect?” (*n* = 248). (B) Plotted survey responses to “On what day after treatment did you get a full affect?” (*n* = 245).

More than half of patients (59%) first noticed the effect of treatment 2 to 3 days after treatment, whereas it took 4 to 7 days to notice the effect in approximately a third of patients (31%) (Figure 1A). The treatment’s complete effect became noticeable on Day 7 for approximately one-third of patients (32%), and for another third (34%), it manifested between Days 8 and 10 (Figure 1B). Only 3% of patients had not yet experienced the full effect at the time of survey completion (7/245; Figure 1B). Of note, 39% of patients noted that the onset of treatment effects seemed to be faster in the forehead, followed by the eye area (21%) and frown lines (17%; Supplemental Figure 3). When asked to choose between prespecified outcomes noted at the time of the complete treatment effect, 46% of respondents felt their faces still looked natural, 20% felt tightness, and 18% felt their faces looked softer (Supplemental Figure 4).

**Figure 2.**
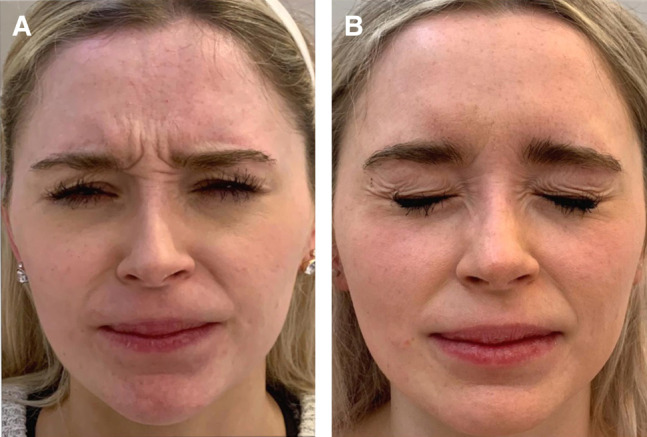
Wrinkle reduction in a 28-year-old female treated with the prabotulinumtoxinA injection into the glabella, forehead, and chin. The patient is shown at baseline (A) and 14 days after treatment (B) at maximum frown. Note the marked reduction in glabellar lines.

**Figure 3.**
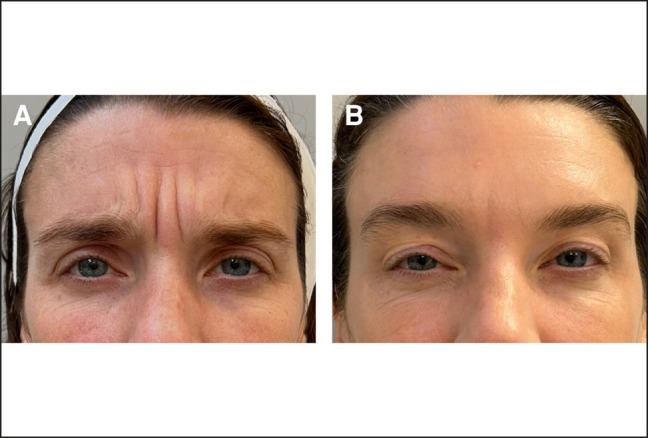
Wrinkle reduction in a 39-year-old female treated with the prabotulinumtoxinA injection into the glabella and forehead. The patient is shown at baseline (A) and 14 days after treatment (B) with the patient frowning/purposeful corrugator. Note the marked reduction in glabellar wrinkles.

Before and after pictures of example cases demonstrate prabotulinumtoxinA injection was effective at reducing wrinkles in the glabellar area (Figures 2, 3).

### Patient Satisfaction

**Figure 4.**
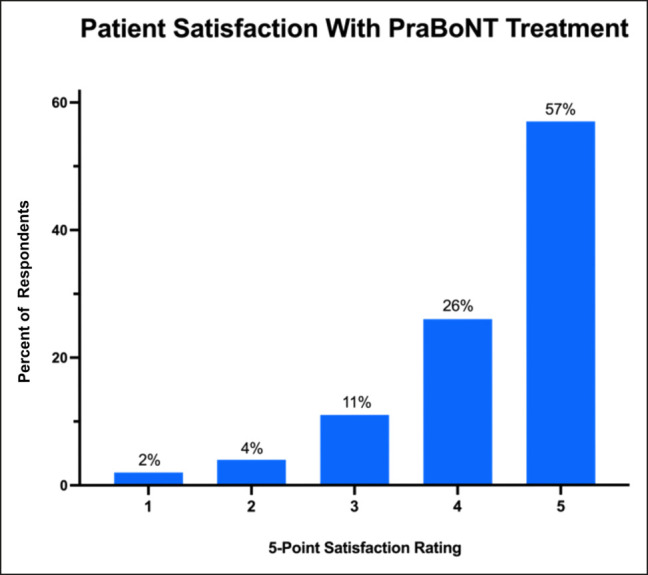
Patient satisfaction with prabotulinumtoxinA treatment rated on a 5-point satisfaction scale.

Among 254 respondents, 83% rated their recent treatment with prabotulinumtoxinA positively as a 4 or higher on a 5-point satisfaction scale (1 = not at all satisfied, 5 = extremely satisfied; Figure 4). In contrast, only 6% rated their treatment negatively as a 2 or lower (Figure 4).

The 5-point satisfaction rating of patients immunized with the COVID vaccine within 6 months of treatment was similar to that of patients receiving the vaccine prior to 6 months before treatment (4.2 ± 1.1 vs 4.4 ± 0.9).

**Figure 5.**
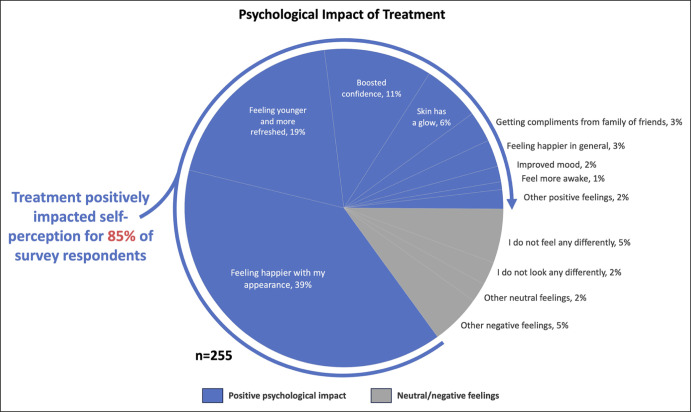
Patients were asked how they saw themselves after treatment using predesignated answers or a customized response, which are denoted here as “other” positive, neutral, or negative feelings.

When patients were asked how they saw themselves after treatment using predesignated answers or a customized response, the 3 most common responses were “feeling happier with appearance” (39%), “feeling younger and more refreshed” (19%), and “having boosted confidence” (11%; Figure 5). Cumulatively, treatment positively impacted self-perception for 85% of survey respondents. In contrast, 10% expressed neutral sentiments, whereas 5% reported negative feelings about their appearance after undergoing treatment.

After receiving the treatment, 72% of respondents would choose prabotulinumtoxinA again (Supplemental Figure 5). Among the 7% who expressed reluctance, the primary reason was dissatisfaction with the treatment, which was sometimes linked to adverse effects (Supplemental Figure 6). Patients responding “maybe” (20%) were often waiting to experience the duration and uniformity of treatment before making a definitive decision. Some patients expressed a desire for more information regarding the safety and effectiveness of prabotulinumtoxinA in comparison with other brands before finalizing their choice.

### Safety

Most patients (67%) experienced no adverse effects following prabotulinumtoxinA injection (Table 1). In the Table 1 below, demographics are shown. Bruising and headaches were the most reported adverse effects, occurring in 10% and 9% of respondents, respectively (Table 1). A minority of patients also reported numbness (4%) and swelling or a lump (4%; Table 1). Soreness, distorted expression, and rash were each reported by 1% to 2% of patients (Table 1).

**Table 1.** Patient-Reported Adverse Effects Following Treatment With PrabotulinumtoxinA

Adverse effect^a^	No. of responses	Percentage (%), *n* = 252
None	169	67
Bruising	26	10
Headaches	24	9
Swelling/Lump	9	4
Numbness	8	3
Distorted expression	4	2
Soreness	3	1
Rash	3	1
Other	6	2

^a^Participants were able to select multiple choices from a provided list.

## DISCUSSION

These real-world data, collected during the first months following prabotulinumtoxinA licensing in the United Kingdom, confirm the safety and efficacy for improvement in the appearance of moderate-to-severe glabellar lines, consistent with the published outcomes of the randomized controlled trials. Importantly, in the presented study, efficacy and satisfaction were observed in a much broader population, including patients over the age of 65 years, patients who have received BoNT-A within the past 12 months, and patients who were treated in multiple areas of the face.

When considering clinical trial metrics, those that capture patient satisfaction and patient perceptions of aesthetic improvement are keys. In this study, 83% of patients completing the survey between 14 and 35 days following treatment were satisfied with their prabotulinumtoxinA treatment, aligning closely with the 89% to 92% of patients satisfied at 14 and 30 days in the Phase III clinical trials.^7,8^ Similarly, 59% of patients first noticed the effect of treatment between 2 and 3 days after treatment, which compares well with clinical trial results demonstrating that 51% to 56% of patients perceived a positive response on the Global Aesthetic Improvement Scale at the Day 2 follow-up visit.^7,8^ In the presented study, 66% of patients reported that the peak effect occurred between postinjection Days 7 and 10. Although not assessed in the Phase III clinical trials, this result aligns with the average time to peak effect of 9.6 days reported in a recent randomized trial, comparing prabotulinumtoxinA and onabotulinumtoxinA for the treatment of lateral canthal rhytids.^13^

The presented study also indicates that prabotulinumtoxinA injection for wrinkle reduction is safe in a broader population and that the side-effect profile is as expected. Adverse effects were experienced by approximately one-third of patients, which is similar to the 38% to 39% of patients experiencing adverse effects in the clinical trials.^7,8^

The real-world study presented here was not designed to directly compare prabotulinumtoxinA with other BoNT-A formulations; however, the results may provide insights into patient preferences and perceptions of product efficacy. Among survey respondents, 93% had previously received an antiwrinkle injection treatment, which would likely have been 1 of the 3 formulations licensed in the United Kingdom at the time, namely onabotulinumtoxinA, incobotulinumtoxinA, or abobotulinumtoxinA. When patients were asked whether they would choose prabotulinumtoxinA again, 72% said yes, and 20% said maybe, highlighting a potential patient preference for prabotulinumtoxinA for their next treatment. For those who indicated “maybe,” many patients indicated that they were waiting to compare the duration of prabotulinumtoxinA with their previous treatments before deciding.

As the primary interface with the surrounding world, human facial appearance and expression have a functional role in interpersonal communication, perceived age, and attractiveness.^14,15^ As individuals age, upper facial lines, such as those in the glabellar area, begin to manifest and progress, becoming some of the most noticeable signs of aging.^15^ Furthermore, the permanence of such lines can lead to a discrepancy between a person's genuine emotions and their interpretation by others.^16,17^ Therefore, it is not surprising that signs of aging can significantly impact an individual's emotional state, overall psychological health, self-perception, and interactions with others.^15,18,19^ Importantly, however, the real-world data presented here demonstrate that while changes to mood may be a part of the treatment experience, most patients selected feeling happier with their appearance (39%) or feeling younger and more refreshed (19%), or boosted confidence (11%) as the chief benefit of treatment, rather than improvement in mood or fewer negative feelings.

The real-world approach of this study adds value in its assessment of a wide variety of patients, ranging from 21 to 77 years in age, some of whom were concurrently undergoing other treatments and receiving medications, reflecting the patient population typically encountered in clinical practice. Over a third of patients were receiving HRT at the time of their prabotulinumtoxinA treatment. As a treatment choice for menopausal patients, these patients are an important group within the real-world population seeking aesthetic care.^20^ In addition to taking medication, real-world patients may have also been recently immunized with the COVID vaccine. A recent study suggested a potential link between COVID vaccine immunization and reduced effectiveness of BoNT-A injections.^21^ Analysis of the survey responses did not establish a correlation between satisfaction score and recent immunization with the COVID vaccine. However, the numbers trended in support of reduced effectiveness, warranting future real-world investigations specifically designed to assess the impact of COVID vaccination on BoNT-A injections.

Real-world studies also permit the analysis of individualized treatment approaches. Achieving optimal outcomes with BoNT-A therapy requires a patient-tailored technique that involves a comprehensive understanding of facial anatomy and individual patient assessment to determine the proper dosage, injection quantity, and precise location for injections.^6,22^ Therefore, practicing physicians may find the availability of real-world data resulting from individualized treatments to be both relevant and beneficial. As patients have not been incentivized to participate, these data represent honest, unbiased feedback that can instill confidence around adopting prabotulinumtoxinA into clinical practice.

In the United Kingdom, BoNT-A is considered a prescription only medication, for which advertising is not permitted and can only be discussed one-on-one with a physician.^23^ As a result, patients have reduced BoNT-A brand awareness. Real-world information, such as the findings presented here, can be a valuable resource for physicians when engaging in discussions with patients about their BoNT-A treatment choices. Moreover, survey responses highlight the importance of having these one-on-one discussions.

The study design of this real-world survey has certain limitations that should be considered when interpreting the data, including absence of a control group and potential patient recall bias. Additional limitations stem from the study's real-world nature. For example, although all patients in this study received injections in the glabellar region, some of them also underwent injections in other areas. Consequently, their reported experiences encompass treatments in these additional regions. Considering that BoNT-A characteristics can vary across different anatomical sites,^24^ the inclusion of additional treatment areas in the analysis group could confound results.

The safety data may also be affected by the real-world nature of patient-reported, uncontrolled data collection. For example, 1 patient mentioned uncertainty regarding whether bruising resulted from prabotulinumtoxinA injection or a different aesthetic procedure received during the same visit. Moreover, patient-reported adverse effects can be difficult to interpret in the absence of physical examination. Although these limitations are inherent to the study, they reflect real-world clinical practice and, therefore, can provide valuable insights for practicing clinicians.

## CONCLUSIONS

In this study, real-world data on prabotulinumtoxinA following its licensing in the United Kingdom validate its safety and effectiveness in improving the appearance of moderate-to-severe glabellar lines in adults. These findings include patient satisfaction and safety consistent with clinical trials, and evidence supportive of a predictable onset and peak effects. Furthermore, 72% of patients indicated that they would choose prabotulinumtoxinA again, supporting good clinical performance for prabotulinumtoxinA in real-world populations. The real-world approach of this study mirrors typical clinical practice, yielding several relevant findings for physicians.

## Supplementary Material

ojae013_Supplementary_Data
