# Supplementary material for: Real-World Patient Experience With PrabotulinumtoxinA in the United Kingdom: A Single-Center Survey and Analysis of 254 Patients
Source: Aesthet Surg J Open Forum. 2024 Feb 27;6:ojae013. doi: 10.1093/asjof/ojae013 (PMC11140816; doi:10.1093/asjof/ojae013)
Supplement: ojae013_Supplementary_Data [file ojae013_Supplementary_Data.zip › Supplementary Figure Legends.pdf]

Supplementary Figure Legends

Figure S1: Visual snapshot to demonstrate the variety of patients evaluated in this real-world study, ranging from 21 to 77 years in age. Baseline photographs are shown.

Figure S2. When was your last anti-wrinkle injection treatment prior to this treatment? *(select one)*

Figure S3. Did you notice any area kick in quicker? *(select one)*

Figure S4. Did you notice any of these outcomes, when you anti-wrinkle injection treatment fully settled in? *(select one)*

Figure S5. Would you be happy to have this brand version of Nuceiva in your next treatment? *(select one)*

Figure S6. Reasons for saying ‘No’ or ‘Maybe’ to using PraBoNT
