# Supplementary material for: Real-World Patient Experience With PrabotulinumtoxinA in the United Kingdom: A Single-Center Survey and Analysis of 254 Patients
Source: Aesthet Surg J Open Forum. 2024 Feb 27;6:ojae013. doi: 10.1093/asjof/ojae013 (PMC11140816; doi:10.1093/asjof/ojae013)
Supplement: ojae013_Supplementary_Data [file ojae013_Supplementary_Data.zip › Supplementary Table 1.pdf]

Supplementary Table 1: PrabotulinumtoxinA Survey Questions

Topic	Questioned Asked
**Previous treatments & medications**	When was your last anti-wrinkle injection treatment prior to this treatment? *(Select one)*<br>Choices include:<br>3 months ago<br>4 months ago<br>5 months ago<br>6 months ago<br>Between 6 months and 1 year<br>This is my first anti-wrinkle injection treatment<br>Other
	Were you taking any medication at the time of injection treatment and if so what medication? *(Long form answer)*
	When did you last have Covid vaccine? *(Date)*
**Experience and effects**	On what day after the treatment did you first notice an effect from Nuceiva? *(Select one)*<br>Choices include:<br>Day 2<br>Day 3<br>Day 4<br>Day 5<br>Day 6<br>Day 7<br>Took up to two weeks<br>Took longer than 14 days<br>I still haven't seen results<br>Other
	On what day after treatment with Nuceiva did you get the full effect? *(Select one)*<br>Choices include:<br>Day 7<br>Day 8<br>Day 9<br>Day 10<br>Day 11<br>Day 12<br>Took up to 13-14 days<br>I still haven't seen results

	Other
	Did you notice any area kick in quicker? *(Select one)*<br>Choices include:<br>Forehead<br>Eye Area<br>Frown Linkes<br>Bunny Lines<br>Chin<br>Other
	Did you notice any of these outcomes when your anti-wrinkle injection with Nuceiva fully settled in? *(Select one)*<br>Choices include:<br>I feel tightness<br>I feel stiffness<br>I felt my face looked softer<br>I felt my face still looked very natural<br>I felt a constricted sensation<br>Other
**Satisfaction and Feedback**	Did you experience any adverse effects from the Nuceiva treatment injections? *(Select all that apply)*<br>Choices include:<br>Prolonged pain<br>Swelling<br>Bruising<br>Numbness<br>Headache<br>Other
	How satisfied were you with your recent treatment of Nuceiva?<br>Rate 1-5; 1= not at all satisfied, 5= extremely satisfied)
	After your treatment with Nuceiva, how do you see yourself? *(Select one)*<br>Choices include:<br>Boosted confidence<br>Motivated to stick to a fitness routine<br>Getting compliments from friends and family<br>Feeling happier with my appearance<br>Feeling happier in general<br>Improved mood<br>Feel more awake

	Feel younger and more refreshed<br>Skin has a glow<br>I do not feel any differently<br>I do not look any differently<br>Other
	Would you be happy to have this brand version of Nuceiva in your next anti-wrinkle injection treatment? *(Select one)*<br>Choices include:<br>Yes<br>No*<br>Maybe<br>Other<br><br>*If you answered no or maybe to the previous question, please could you provide us with a reason.
	Any other feedback? *(Long form answer)*
