# Supplementary figures and images for: Real-World Patient Experience With PrabotulinumtoxinA in the United Kingdom: A Single-Center Survey and Analysis of 254 Patients
Source: Aesthet Surg J Open Forum. 2024 Feb 27;6:ojae013. doi: 10.1093/asjof/ojae013 (PMC11140816; doi:10.1093/asjof/ojae013)

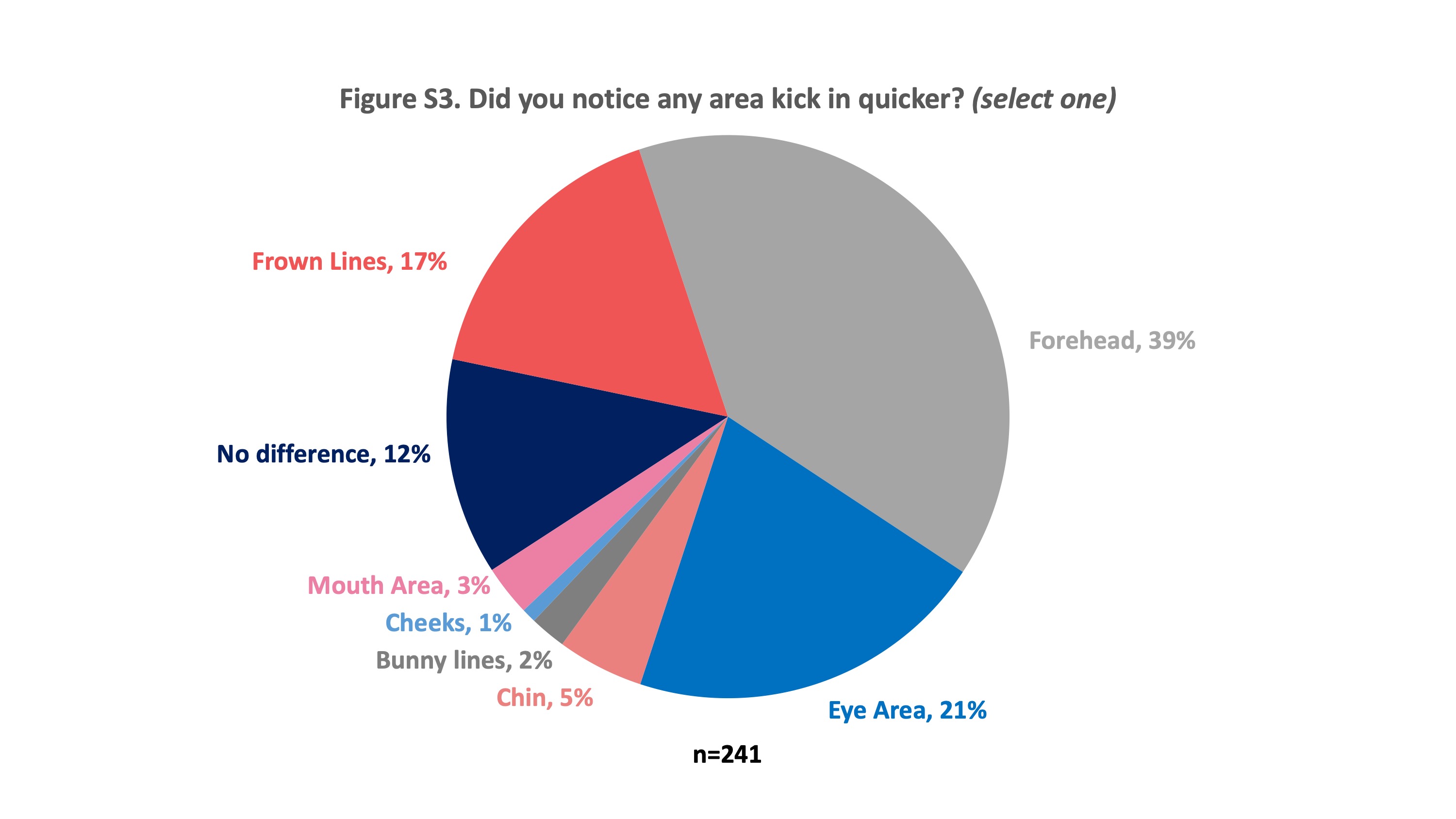

Supplement: ojae013_Supplementary_Data [file ojae013_Supplementary_Data.zip › Supplementary Figure 3_REV.jpg]

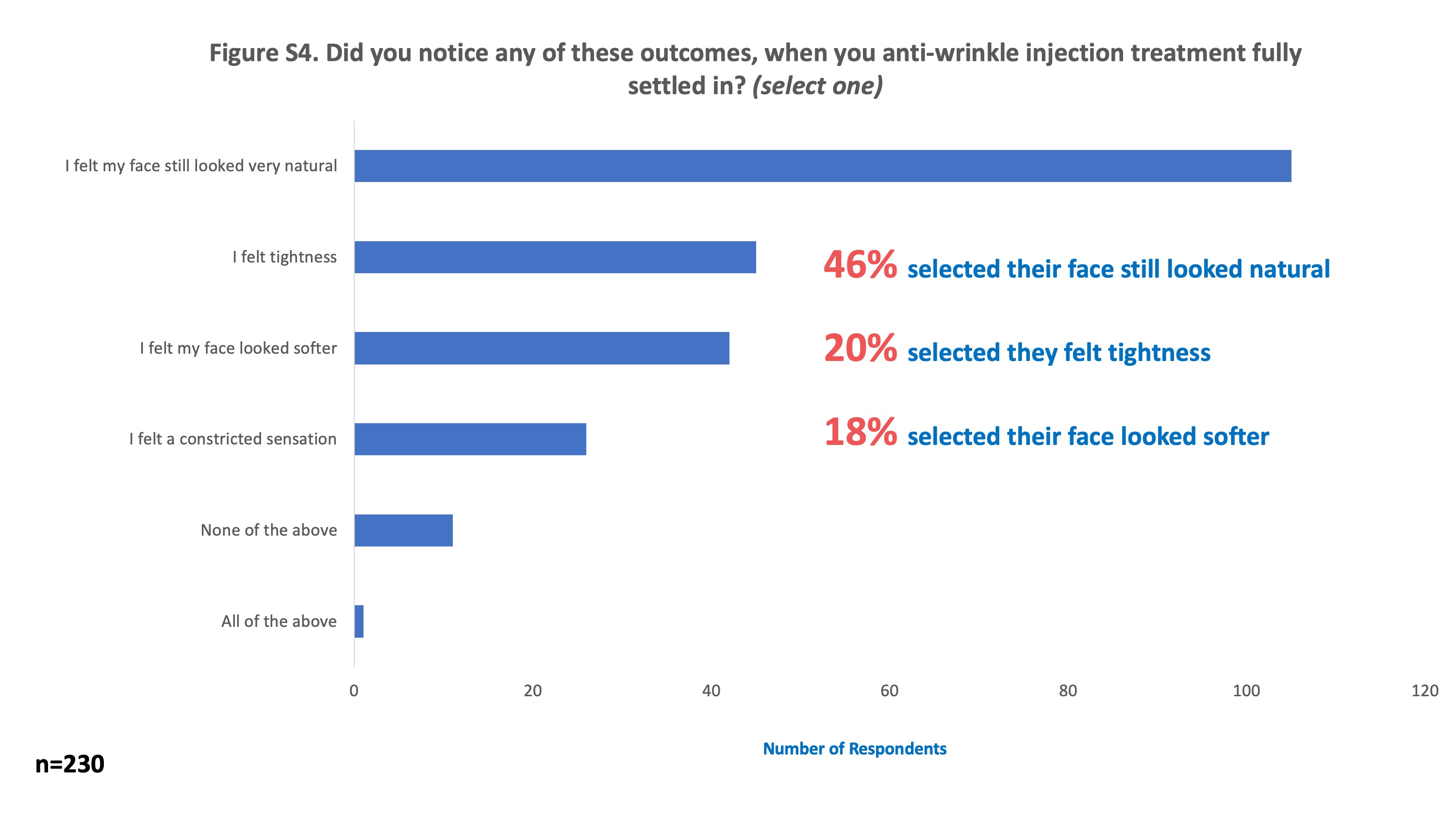

Supplement: ojae013_Supplementary_Data [file ojae013_Supplementary_Data.zip › Supplementary Figure 4_REV.jpg]

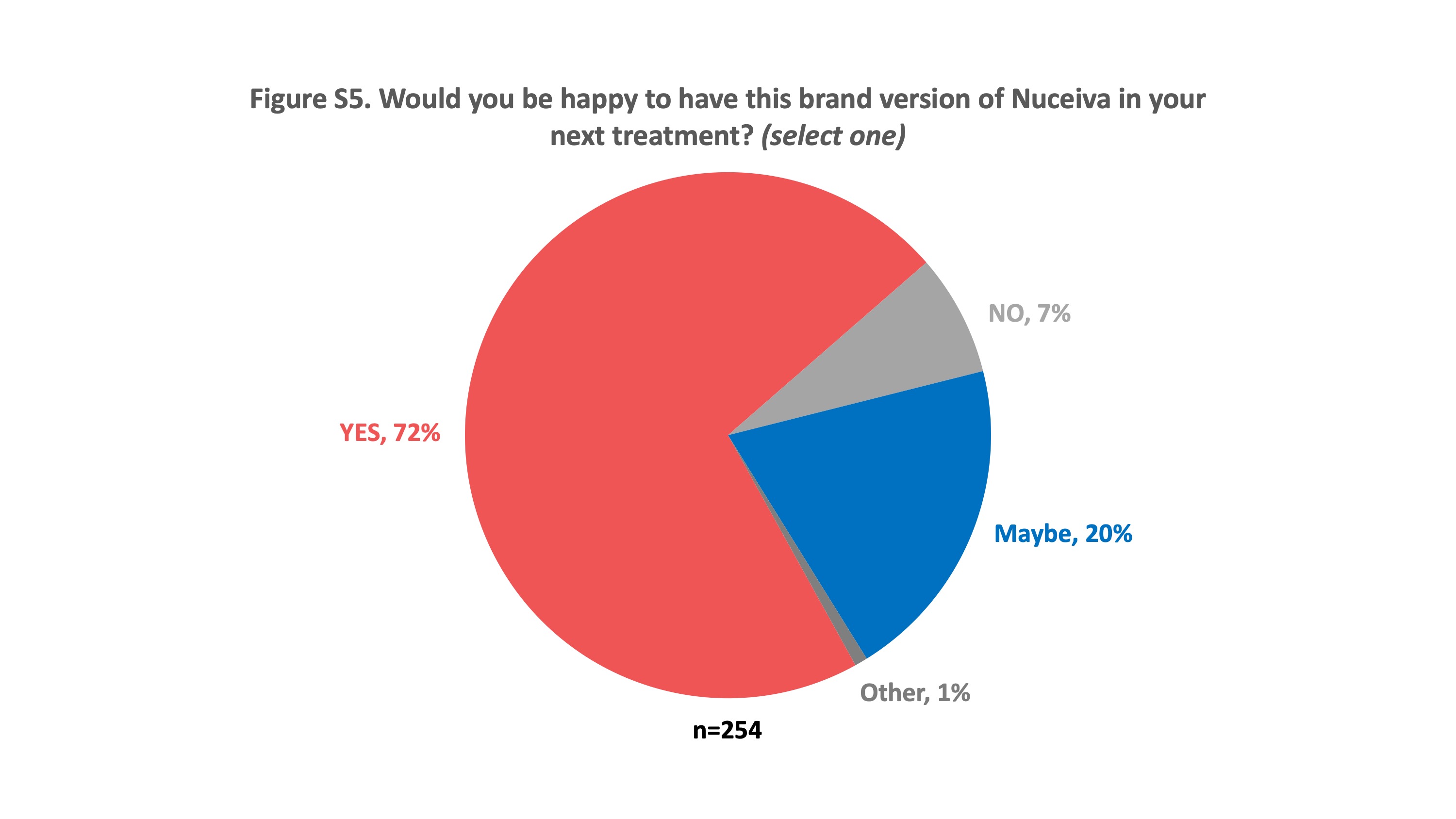

Supplement: ojae013_Supplementary_Data [file ojae013_Supplementary_Data.zip › Supplementary Figure 5_REV.jpg]

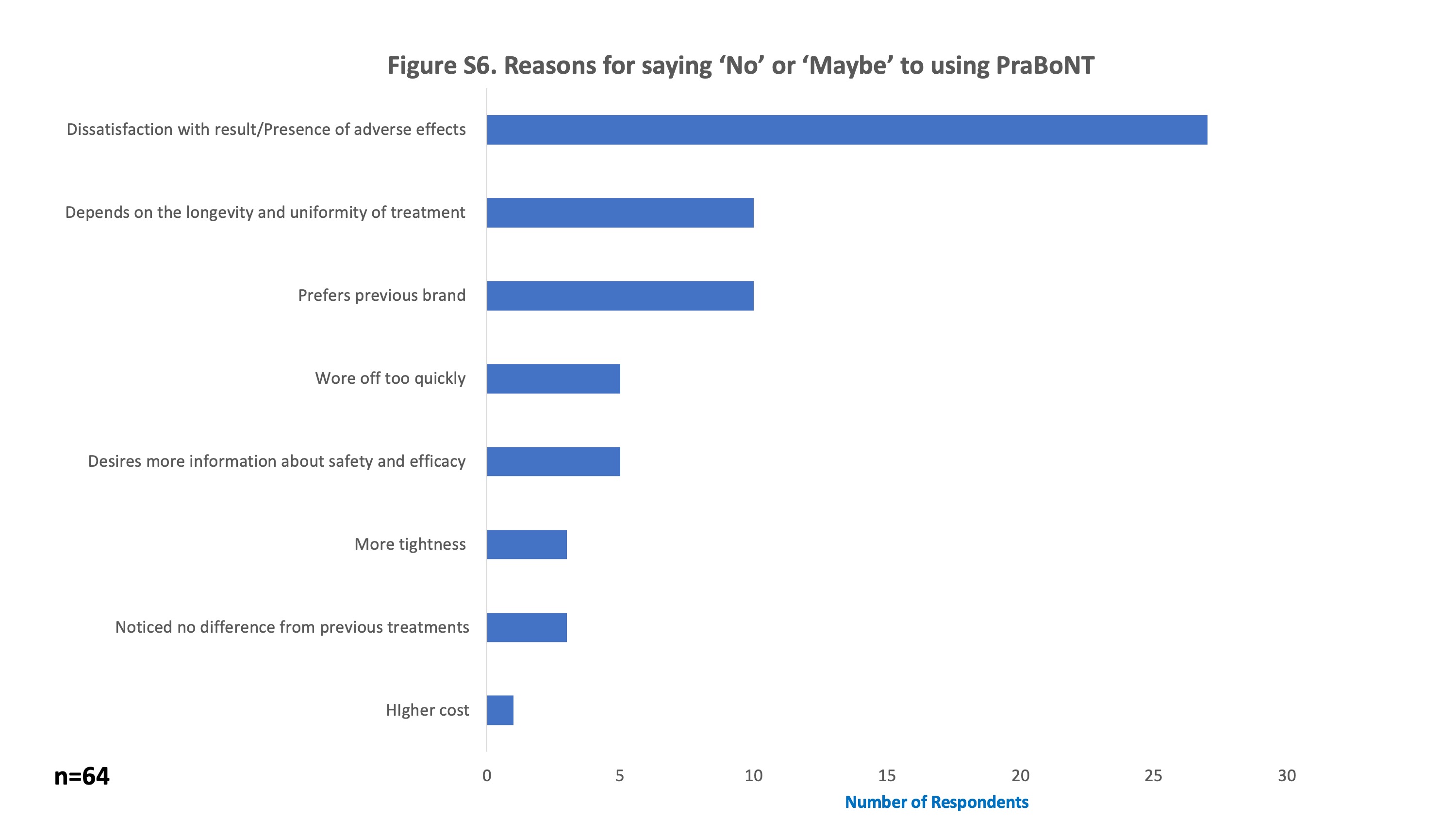

Supplement: ojae013_Supplementary_Data [file ojae013_Supplementary_Data.zip › Supplementary Figure 6_REV.jpg]

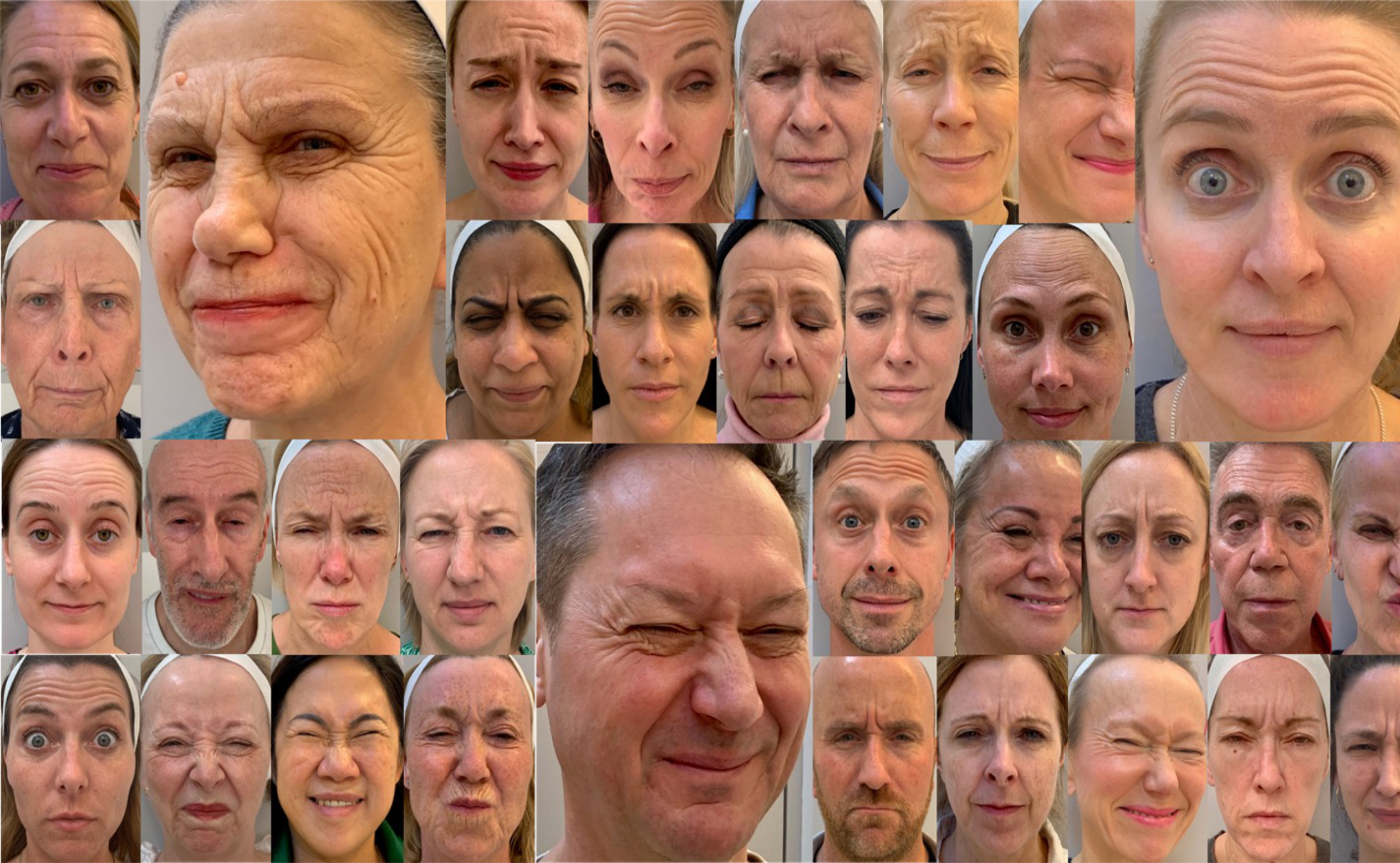

Supplement: ojae013_Supplementary_Data [file ojae013_Supplementary_Data.zip › Supplementary Figure 1_REV.jpg]

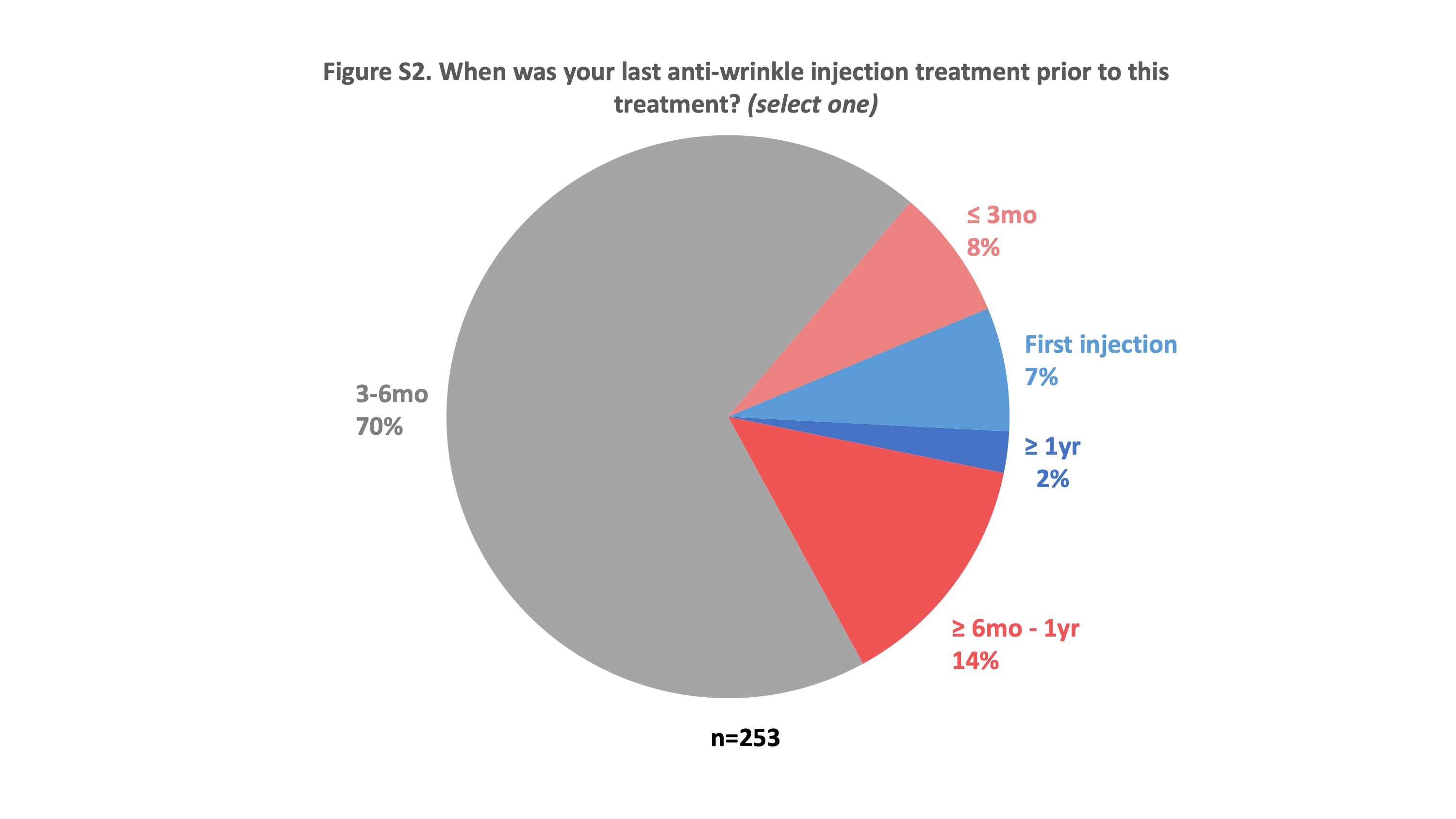

Supplement: ojae013_Supplementary_Data [file ojae013_Supplementary_Data.zip › Supplementary Figure 2_REV.jpg]
